# Supplementary material for: Structural remodeling of the mitochondrial protein biogenesis machinery under proteostatic stress
Source: Sci Adv. 2026 Mar 4;12(10):eaed3579. doi: 10.1126/sciadv.aed3579 (PMC12959407; doi:10.1126/sciadv.aed3579)
Supplement: Supplementary file 1 — Figs. S1 to S9 Tables S1 to S3 [file sciadv.aed3579_sm.pdf]

Science Advances
AAAS

Supplementary Materials for

Structural remodeling of the mitochondrial protein biogenesis machinery under proteostatic stress

Kenneth Ehses *et al.*

Corresponding author: Iban Ubarretxena-Belandia, ivan.ubarrechena@ehu.eus;
Rubén Fernández-Busnadiego, ruben.fernandezbusnadiego@med.uni-goettingen.de

Sci. Adv. **12**, eaed3579 (2026)
DOI: 10.1126/sciadv.aed3579

This PDF file includes:

Figs. S1 to S9
Tables S1 to S3

Fig. S1.

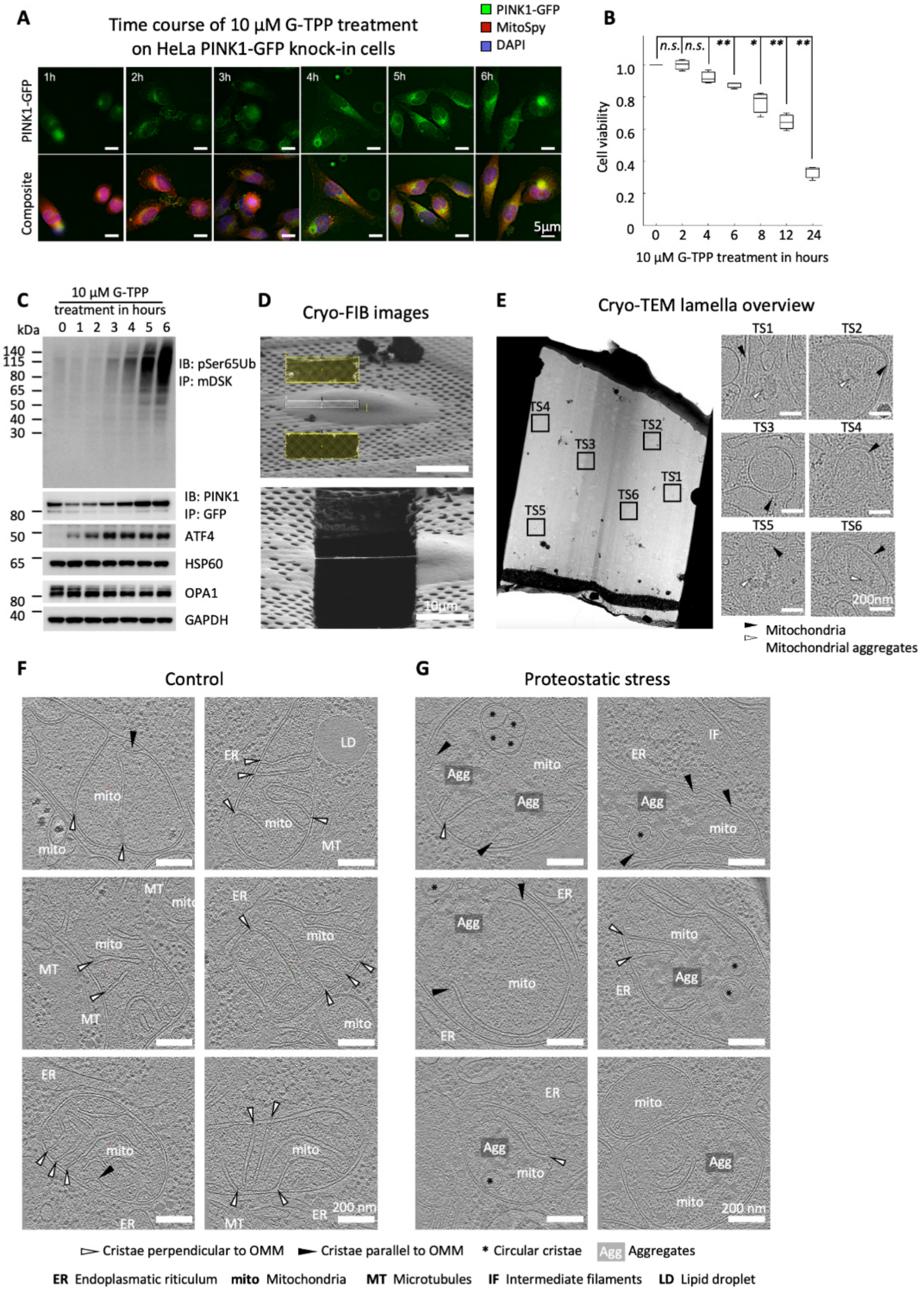

G-TPP treatment induces proteostatic stress in HeLa cells. (**A**) Fluorescence microscopy of PINK1-GFP HeLa cells at time intervals following 10 µM G-TPP treatment. Increasing co-localization of PINK1-GFP with the mitochondrial marker MitoSpy indicates PINK1 stabilization at mitochondria upon proteostatic stress. (**B**) Quantification of cell viability as a function of time following 10 µM G-TPP treatment. For each box, the central line indicates the median, while the box boundaries represent 25th and 75th percentiles. Whiskers extend to the most extreme data points not considered outliers. Statistical significance of pairwise comparisons was assessed using a two-sample t-test and indicated by: n.s. ($p > 0.05$), * ($p < 0.05$) and ** ($p < 0.01$). N = 3 independent experiments. (**C**) Western blot analysis of the effects of G-TPP

treatment on PINK1 accumulation and activation, measured by increased levels of ubiquitin phosphorylation at serine 65 (pSer65Ub). OPA1 band shifts and ATF4 activation serve as additional indicators of mitochondrial stress. (**D**) Ion beam-induced secondary electron images of a HeLa cell vitrified on an EM grid, shown during (top) and after (bottom) cryo-FIB milling. (**E**) Left: cryo-TEM overview of a cryo-FIB-milled lamella from a vitrified HeLa cell that underwent 10 μM G-TPP treatment. Boxed regions were selected for tomographic tilt series (TS) acquisition. Right: Magnified views of the regions of tomographic data acquisition highlighting mitochondria (black arrowheads). In some cases, mitochondrial aggregates (white arrowheads) were readily distinguishable in lamella overviews. (**F–G**) Gallery of tomographic slices from untreated control (F) and G-TPP–treated cells under proteostatic stress (G). Cellular structures are annotated according to the legend at the bottom of the figure.

Fig. S2.

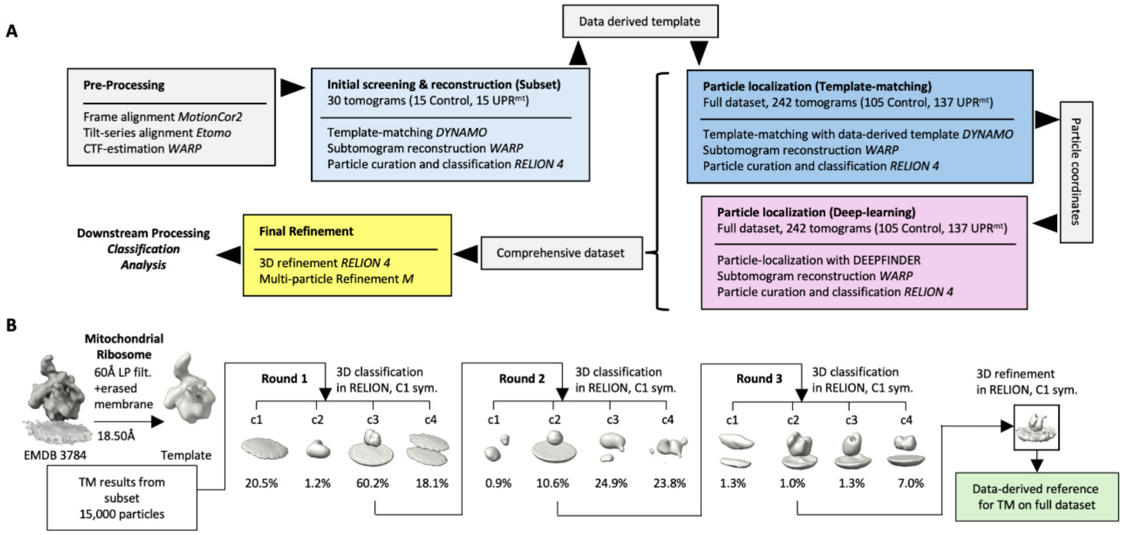

Cryo-ET particle picking workflow and localization of mitochondrial complexes by template matching. (**A**) Schematic overview of the processing strategy for mitochondrial ribosomes and mHsp60 complexes. After initial preprocessing, mitochondrial complexes were identified and their relative abundance assessed by template matching in DYNAMO and subtomogram averaging in RELION 4.0 on a subset of data. Following successful reconstruction, the resulting density maps were used as templates for template matching on the full datasets. Curated particle coordinates were then input into DEEPFINDER for deep-learning–based particle detection to improve accuracy and coverage. The combined datasets from both approaches were refined in RELION 4.0 and M to generate final density maps and particle orientations for downstream analyses. (**B**) Classification steps for mitochondrial ribosome complexes during initial screening. The reference map was low-pass filtered to 60 Å resolution and rescaled to 18.5 Å pixel size to match the dimensions of the tomogram. All classification steps were performed without imposing symmetry. Abbreviations: C1 sym, C1 symmetry; LP filt, low-pass filtered; TM, template matching.

Fig. S3.

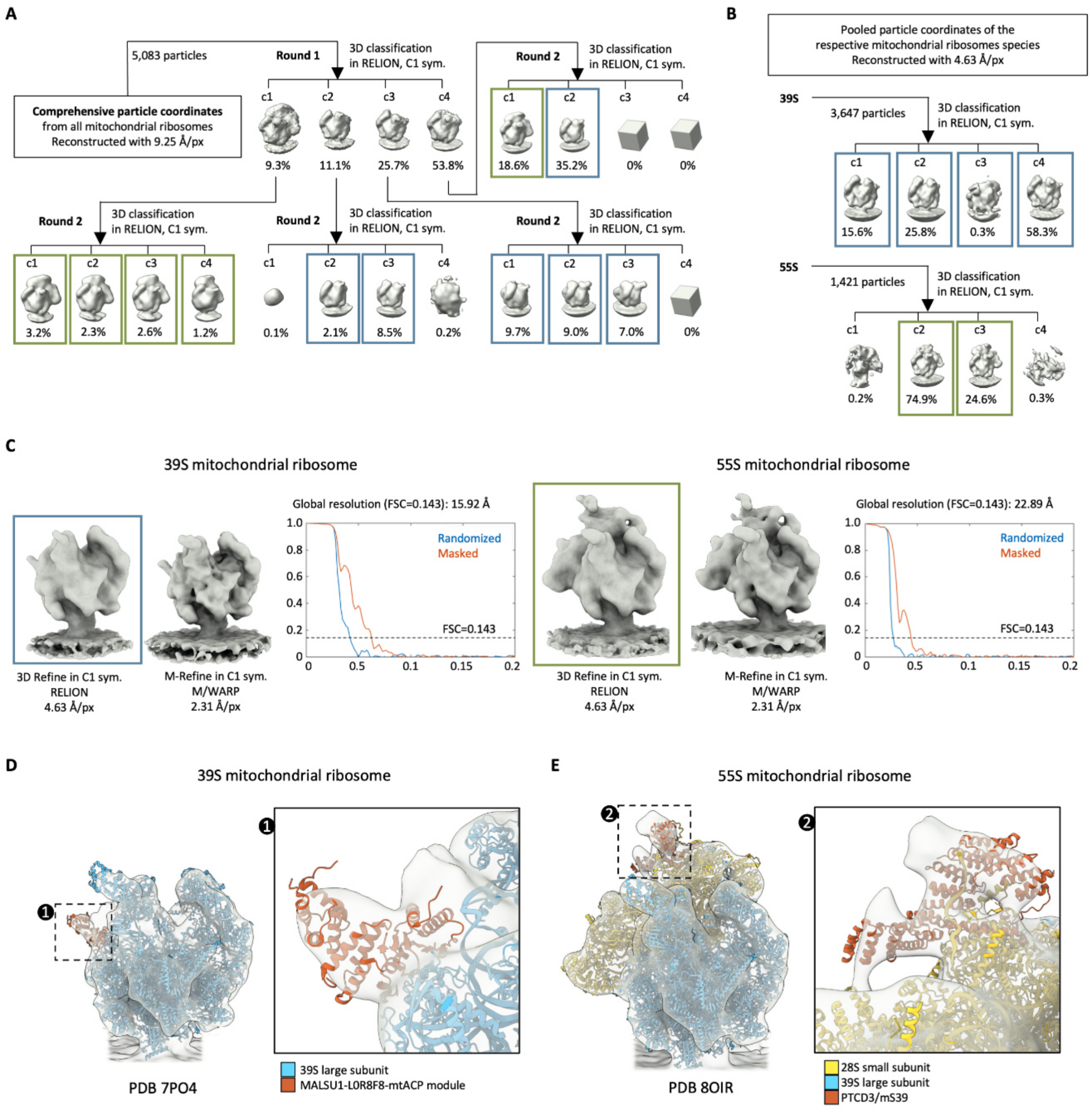

***In situ* structural determination of mitochondrial ribosomes.** (**A**) Curated and distance-filtered particle coordinates were pooled and reclassified at bin 4 to identify the different assemblies. (**B**) Each assembly was further classified at bin 2 to remove remaining false positives and obtain the final particle sets used for refinement. (**C**) Final subtomogram averaging density maps were obtained after refinement at bin 2 in RELION 4.0, followed by multi-particle refinement at bin 1 using M. Abbreviations: C1 sym, C1 symmetry; FSC, Fourier shell correlation; px, pixel. (**D, E**) Docking of mitochondrial ribosome atomic models into *in situ* subtomogram averaging density maps (shown semitransparent). (D) Docking of the atomic model of a 39S mitochondrial ribosome assembly intermediate (PDB 7PO4). The inset highlights density consistent with the binding of the MALSU1–L0R8F8–mtACP module (*43-46*). (E) Docking of the atomic model of a fully assembled 55S mitochondrial ribosome (PDB 8OIR).

The inset indicates density consistent with the binding of the RNA-binding and translation-regulating PTCD3/mS39 subunit (*47, 48*).

Fig. S4.

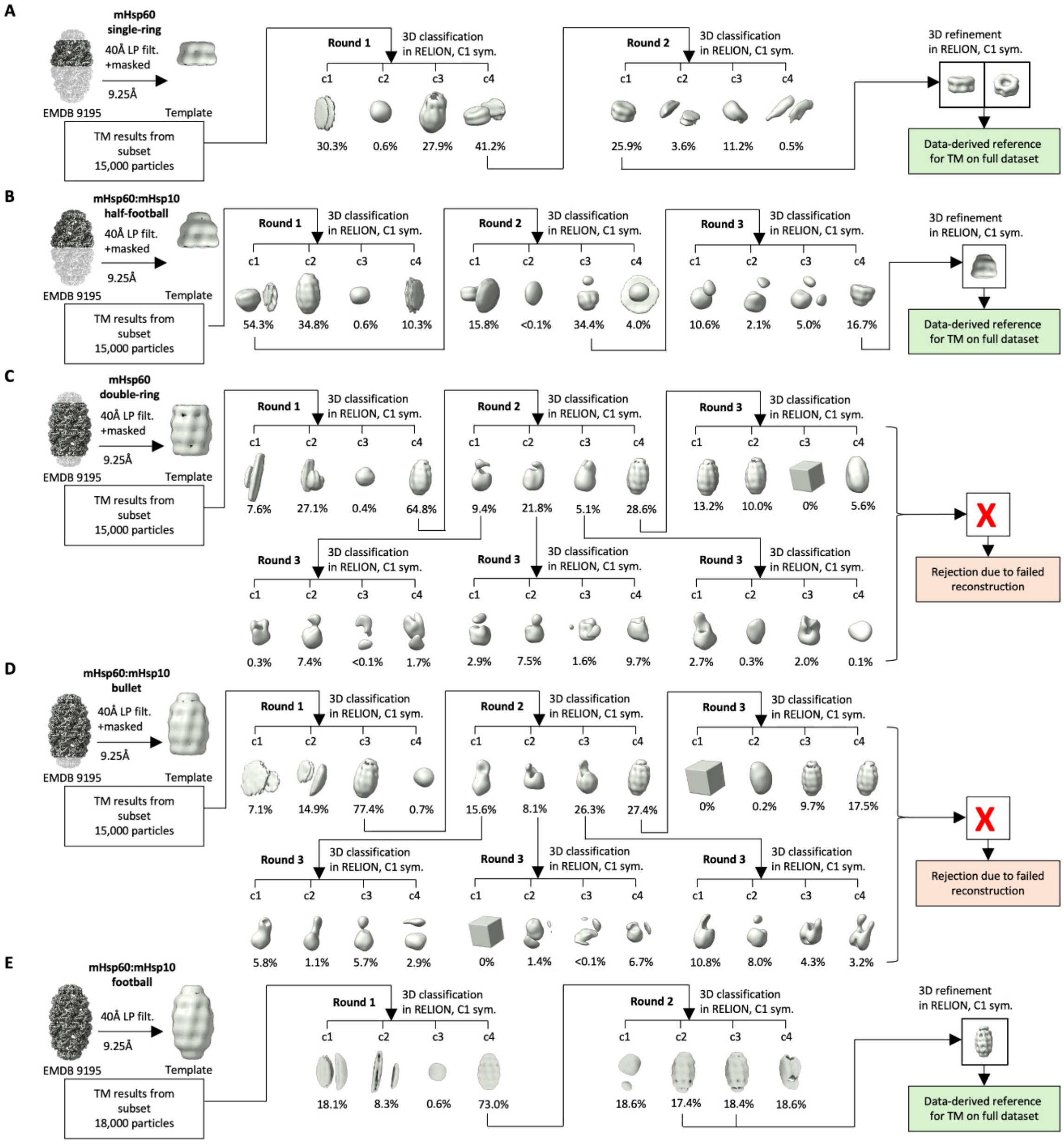

Localization of mHsp60 complexes by template matching. (**A-E**) Classification steps for mHsp60 complexes during initial screening. To detect the presence of various mHsp60 complexes, the single-particle cryo-EM density map of a human mHsp60:mHsp10 football complex (EMDB 9195) was masked to generate references of the different complexes for initial classification. Upon masking, the reference maps were low-pass filtered to 40 Å resolution and rescaled to 9.25 Å pixel size to match the dimensions of the tomogram. All classification steps were performed without imposing symmetry. Abbreviations: C1 sym, C1 symmetry; LP filt, low-pass filtered; TM, template matching.

Fig. S5.

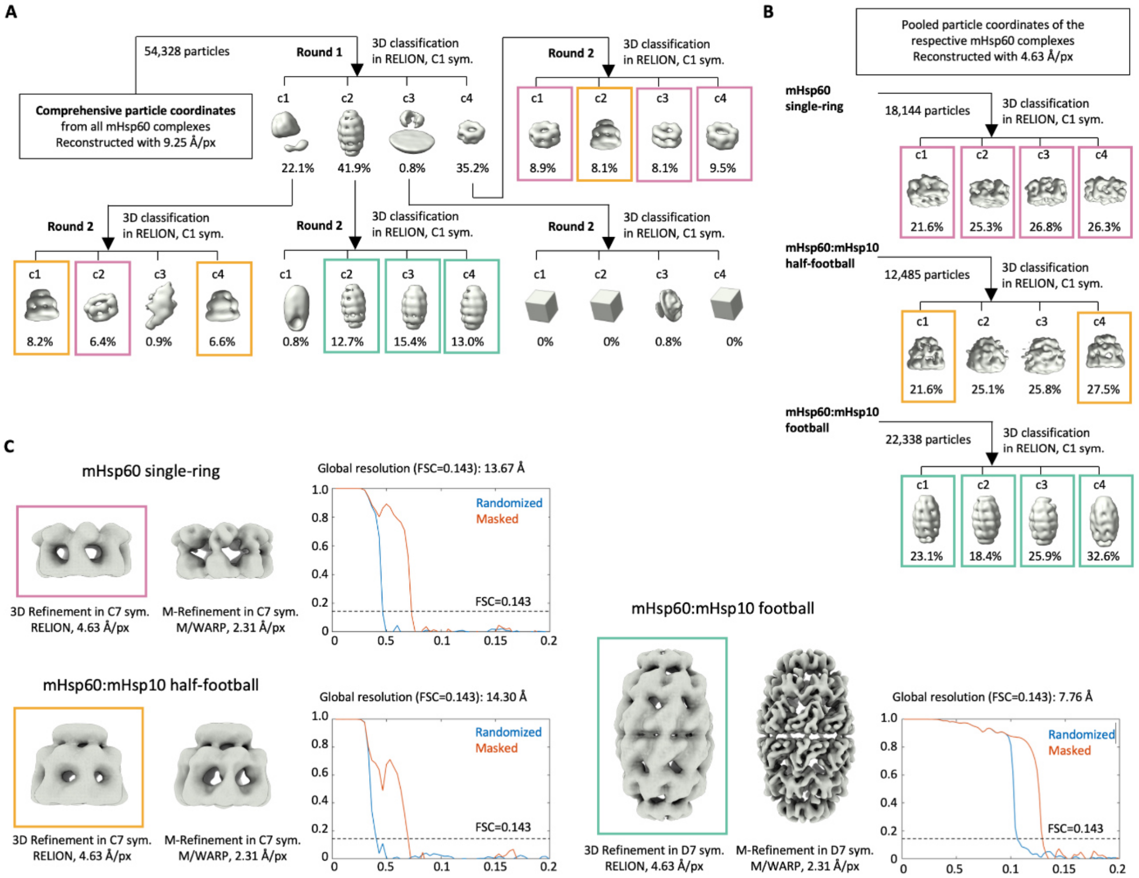

***In situ* structural determination of mHsp60 complexes.** (**A**) Curated and distance-filtered particle coordinates were pooled and reclassified at bin 4 to identify the different assemblies. (**B**) Each assembly was further classified at bin 2 to remove remaining false positives and obtain the final particle sets used for refinement. (**C**) Final subtomogram averaging density maps were obtained after refinement at bin 2 in RELION 4.0, followed by multi-particle refinement at bin 1 using M. Abbreviations: C1 sym, C1 symmetry; FSC, Fourier shell correlation; px, pixel.

Fig. S6.

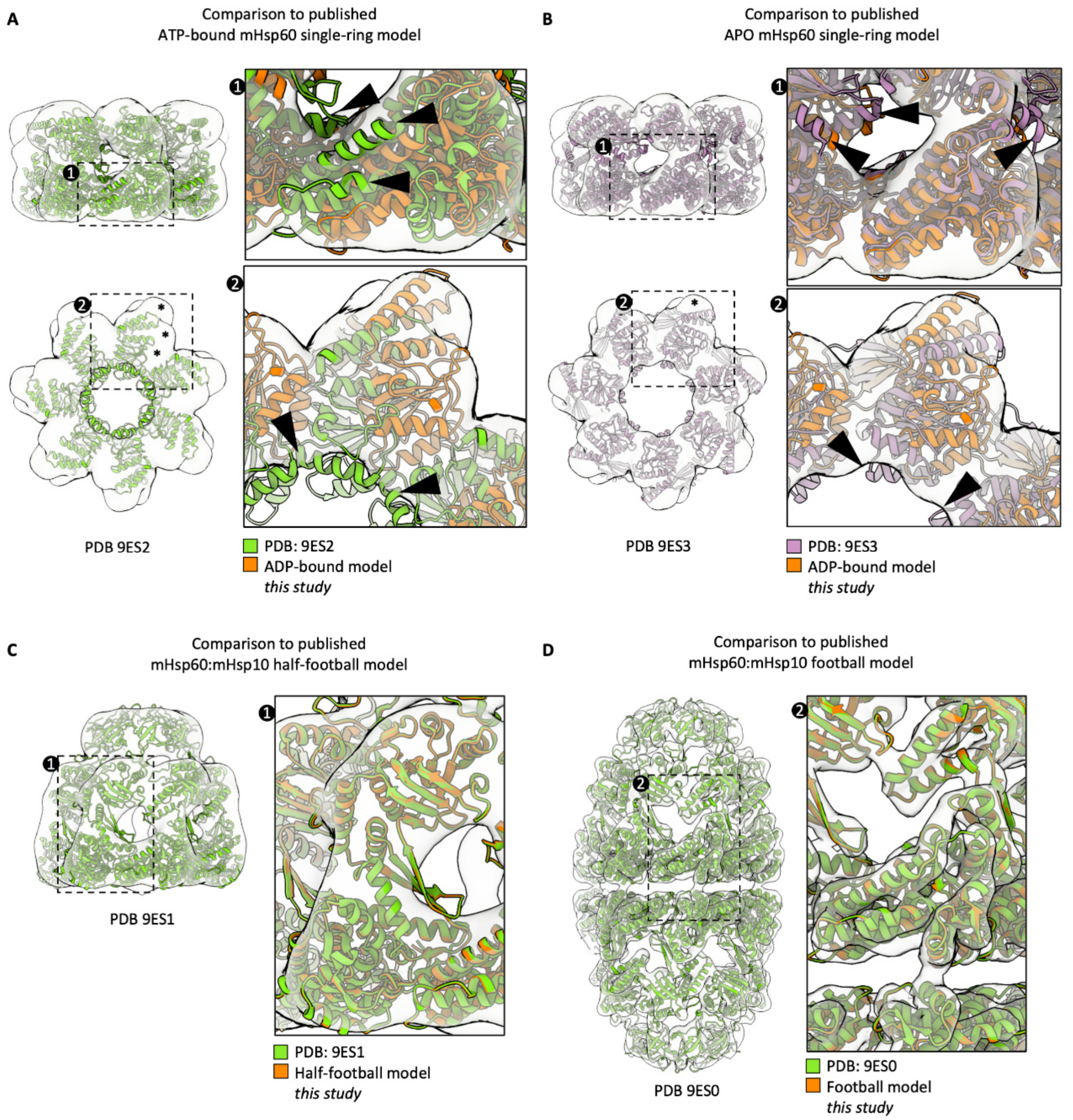

Docking of mHsp60 atomic models into subtomogram averaging density maps. In all cases, *in situ* subtomogram averaging maps are shown semitransparent. (**A–D**) Docking of atomic models of WT mHsp60 complexes derived from single-particle cryo-EM into subtomogram averaging maps determined *in situ* by cryo-ET. We further compare the cryo-EM structures determined in this study (orange) with published structures of mHSp60 complexes (green, purple). For clarity, comparisons are only shown with published structures from (*55*), which are representative of most other published mHps60 structures (Table S 1). (A, B) Visual comparison of the fit into subtomogram maps of ATP-bound (A; PDB 9ES2) and apo (B; PDB 9ES3) mHsp60 single-ring structures against ADP-bound mHsp60 (this study). Regions where the docked ATP and apo structures do not fit well the subtomogram density map are indicated by black arrowheads (structures extending beyond the map) and stars (densities in the map unaccounted for by the structures). See also Table S 3 for quantitative analysis of the fits by

MDFF. (C, D) Overlays of published mHsp60:mHsp10 structures indicate only minimal variations compared to the ones determined in this study (see also Table S 1).

Fig. S7.

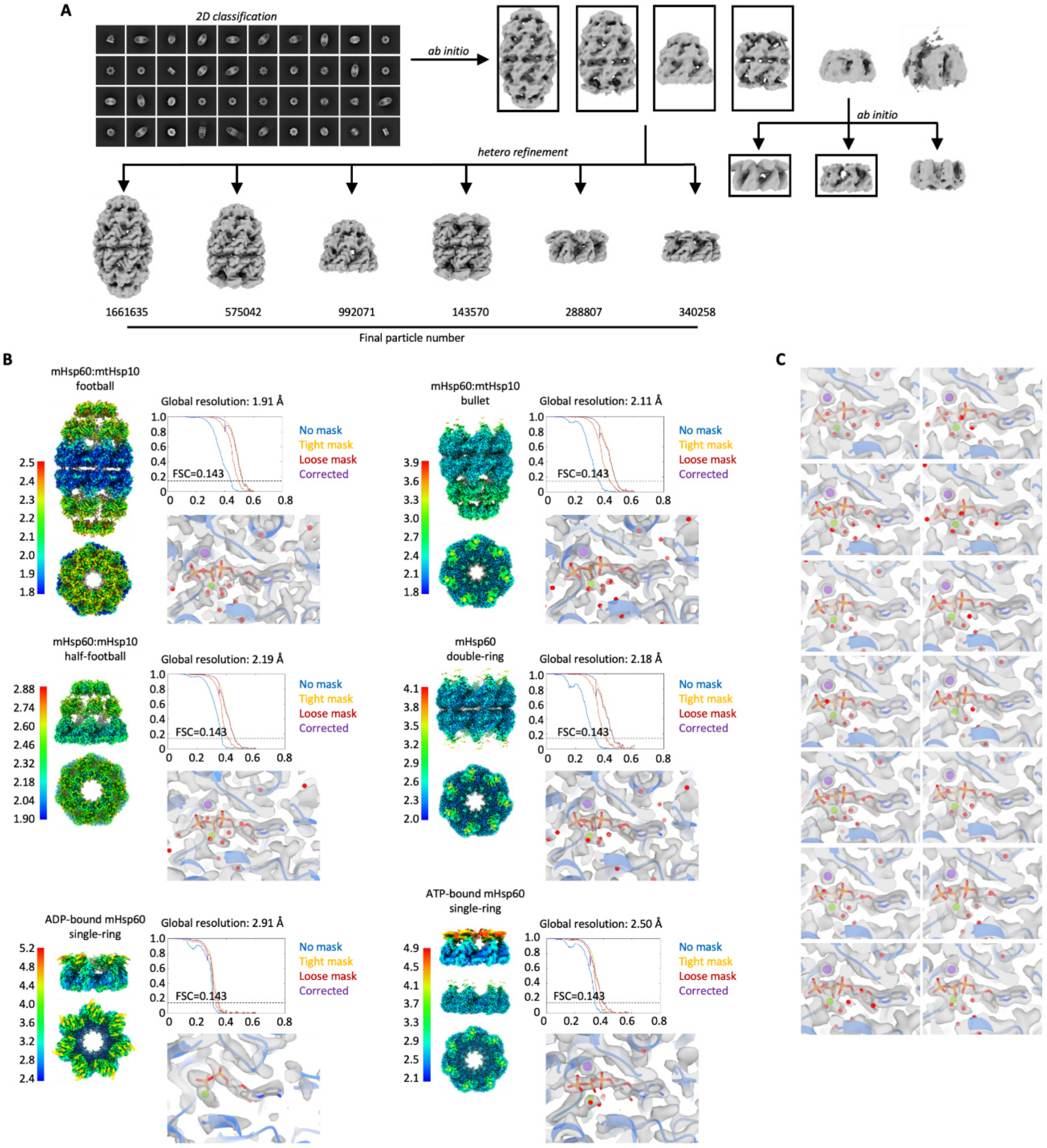

Structures of mHsp60 complexes determined *in vitro* by single-particle cryo-EM. (**A**) Cryo-EM data processing workflow for mHsp60 complexes. All datasets were processed independently, starting with particle picking, iterative 2D classification, and *ab initio* reconstruction. An additional *ab initio* reconstruction was performed specifically for particles contributing to single-ring classes. Boxes mark the *ab initio* volumes used for heterogeneous refinement. (**B**) Cryo-EM maps of ATP-bound mHsp60:mHsp10 football (1.9 Å nominal resolution), ATP-bound mHsp60:mHsp10 half-football (2.2 Å nominal resolution), ATP-bound mHsp60:mHsp10 bullet (2.1 Å nominal resolution), ADP-bound mHsp60 single-ring (2.9 Å nominal resolution), ATP-bound mHsp60 double-ring (2.2 Å nominal resolution), and ATP-bound mHsp60 single-ring (2.5 Å nominal resolution) colored as a function of local resolution

displayed as side and top views. FSC curves and detailed views of the nucleotide-binding sites for each species are also shown, displaying overlays of the density map (semitransparent) fitted with atomic models. The protein is depicted as blue ribbon, nucleotides as sticks, and Mg^{2+} ions (green), K^{+} ions (purple) and water molecules (red) as spheres. Cryo-EM maps were refined with D7 symmetry for the football and double-ring complexes, and C7 symmetry for the half-football, bullet, single-ring assemblies. For the ATP-bound mHsp60 single-ring, the map is also shown upon gaussian-filtering (top) to enable visualization of the highly-dynamic apical domains. (**C**) Nucleotide-binding sites of all 14 subunits of the ATP-bound mHsp60:mHsp10 football complex obtained by refinement without imposing symmetry (C1 symmetry). Abbreviations: FSC, Fourier shell correlation.

Fig. S8.

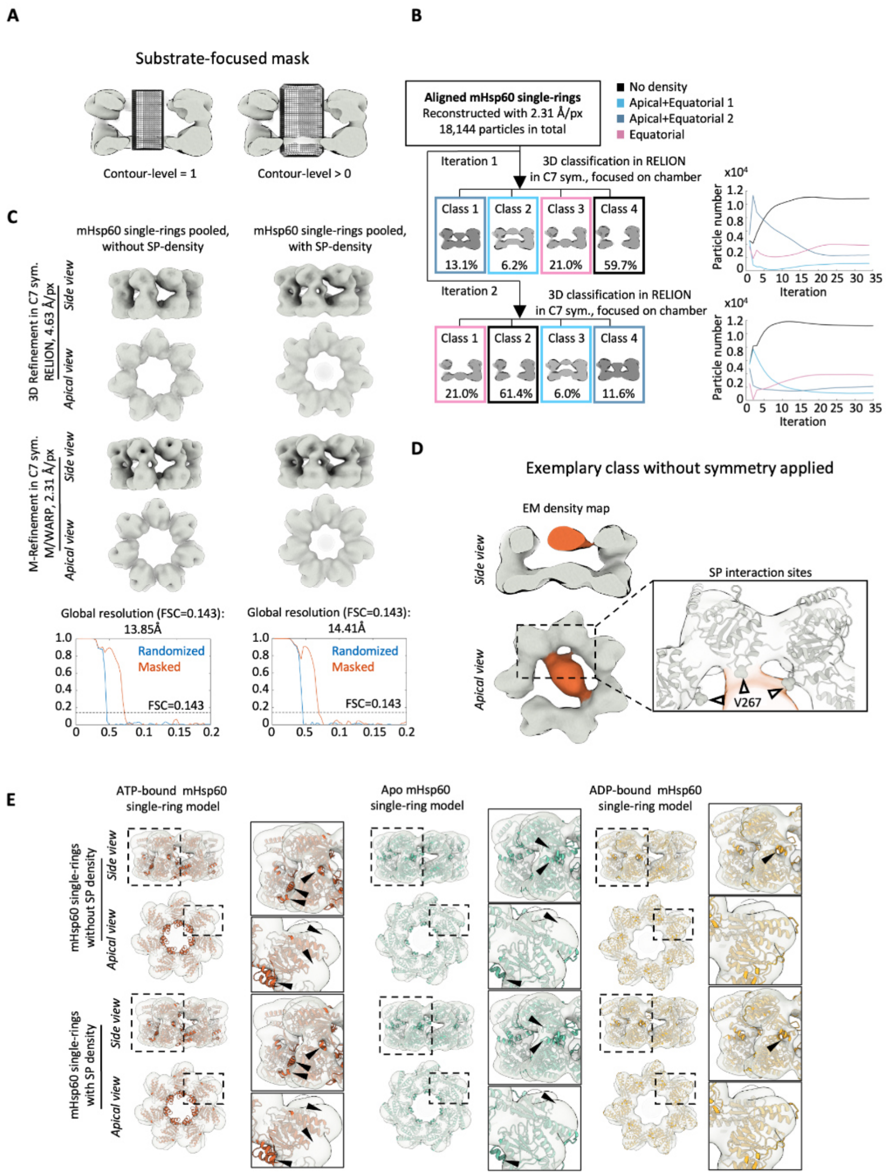

Substrate protein classification of mHsp60 single-ring complexes. (**A**) Mask of the central cavity used for focused 3D classification shown at different contour levels. The rendering at contour level = 1 represents the mask without the soft edge, while levels >0 visualize the full extent of the mask including the soft-edged boundary. (**B**) Classification results (left) and class occupancies over classification iterations (right) for two independent runs. (**C**) Independent refinements of the "No density" class and all SP-bound classes to assess potential structural differences. (**D**) Exemplary SP-bound class obtained from focused chamber-focused 3D classification in C1. The inset highlights the interactions between the SP density and V267 residues of mHsp60 (shown as spheres and marked by white arrowheads), as also observed in the classification using C7 symmetry (Fig. 5B). (**E**) Docking of mHsp60 single-ring atomic models in different nucleotide states into the final subtomogram averaging maps from (C). Insets show magnified regions of interest, with black arrowheads indicating discrepancies between the model

and the density map. See also Table S 3 for quantitative analysis of the fits by MDFF. Abbreviations: FSC, Fourier shell correlation; px, pixel.

Fig. S9.

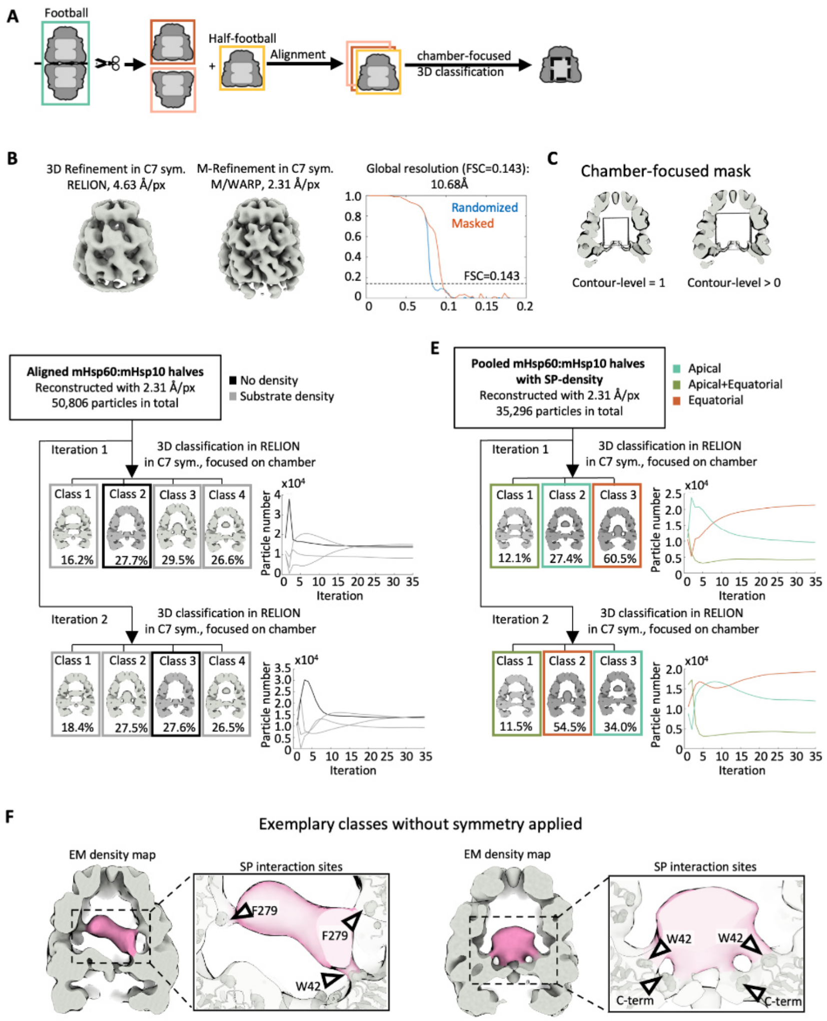

Substrate protein classification of mHsp60:mHsp10 complexes. (**A**) Schematic overview of the processing workflow. mHsp60:mHsp10 football complexes were computationally bisected and their halves were aligned to *bona fide* mHsp60:mHsp10 half-football complexes prior to classification. (**B**) Refinement results for pooled mHsp60:mHsp10 half-maps including both *bona fide* mHsp60:mHsp10 half-footballs and bisected footballs. (**C**) Mask of the central cavity at different contour levels used for focused 3D classification. The rendering at contour level = 1 represents the mask without the soft edge, while levels >0 visualize the full extent of the mask including the soft-edged boundary. (**D**) First round of classification identifying empty chambers. Class averages (left) and class occupancies across iterations (right) are shown for two independent runs. (**E**) Second round of classification identifying different SP localization patterns. Class averages (left) and class occupancies across iterations (right) shown for two independent runs. (**F**) Examples of two different SP-bound classes obtained via focused 3D classification in C1. Insets highlight mHsp60 residues interacting with SPs are shown as spheres and indicated by arrowheads. These interaction sites were also identified using classification with applied symmetry (Fig. 6B).

Table S1.

Reference model	Matching model	RMSD (all pairs; Å)
9ES0 (ATP football)	ATP football (this study)<br>8G7N<br>6MRC	0.3<br>1.8<br>0.6
9ES1 (ATP half-football)	ATP half-football (this study)<br>8G7O<br>6MRD	0.4<br>1.4<br>0.7
9ES2 (ATP double-ring)	ATP single-ring (this study)<br>ADP single-ring (this study)<br>8G7M	1.3<br>11.6<br>4.0
9ES3 (Apo single-ring)	ATP single-ring (this study)<br>ADP single-ring (this study)<br>8G7K<br>7AZP<br>7L7S	9.2<br>6.8<br>1.5<br>0.6<br>1.1

RMSD comparison between published single-particle cryo-EM structures of mHsp60 complexes and those determined in this study. Root mean square deviation (RMSD) calculations were performed using MAXIMOBY/MOBY, considering all common Cα atoms between the models for alignment and RMSD calculation. The structures of WT mHsp60 complexes recently published by Tascón et al. (55) were used as reference (9ES0, ATP-bound football; 9ES1, ATP-bound half-football; 9ES2, ATP-bound double-ring; 9ES3, Apo single-ring). Only one ring of the ATP-bound mHsp60 double-ring 9ES2 model was used for the calculations. The following structures of the V72I mHsp60 mutant were published by Braxton et al. (49): 8G7N (ATP-bound football), 8G7O (ATP-bound half-football), 8G7M (ATP-bound single-ring) and 8G7K (apo single-ring). The following structures of WT mHsp60:mHsp10 were published by Gomez-Llorente et al. (11): 6MRC (ADP-bound football), 6MRD (ADP-bound half-football). The following structures of WT mHsp60 apo single-ring were published by Klebl et al. (50) [7AZP] and Wang&Chen (53) [7L7S].

Table S2.

	ATP-bound mHsp60:mHsp10 football (EMDB 54898) (PDB 9SHG)	ATP-bound mHsp60:mHsp10 bullet (EMDB 54899) (PDB 9SHH)	ATP-bound mHsp60:mHsp10 half-football (EMDB 54900) (PDB 9SHI)
**Data collection and processing**			
Magnification	105000	105000	105000
Voltage (kV)	300	300	300
Electron exposure (e–/Å^2)	50	50	50
Defocus range (μm)	0.8-1.6	0.8-1.6	0.8-1.6
Pixel size (Å)	0.8238	0.8238	0.8238
Symmetry imposed	D7	C7	D7
Initial particle images (no.)	12338206	12338206	12338206
Final particle images (no.)	1661635	575042	992071
Map resolution (Å)	1.91	2.11	2.19
FSC threshold	0.143	0.143	0.143
Map resolution range (Å)	1.845 - 20.712	1.881 - 30.220	1.818 - 25.245
**Refinement**			
Initial model used (PDB code)	9ES0	9ES1/9ES2	9ES1
Model resolution (Å)	2.0	2.2	2.5
FSC threshold	0.5	0.5	0.5
Map sharpening *B* factor (Å^2)	-60.4	-60.3	-75.5
Model composition			
Non-hydrogen atoms	68854	63040	34531
Protein residues	8778	8078	4389
Ligands	42	42	21
B factors (Å^2)			
Protein	43.14	69.33	47.69
Ligand	11.32	17.88	18.59
R.m.s. deviations			
Bond lengths (Å)	0.004	0.004	0.004
Bond angles (°)	0.680	0.676	0.636
Validation			
MolProbity score	1.05	1.20	1.10
Clashscore	2.65	4.21	3.13
Poor rotamers (%)	0.00	0.00	0.00
Ramachandran plot			
Favored (%)	98.73	98.36	98.62
Allowed (%)	1.27	1.64	1.38
Disallowed (%)	0.00	0.00	0.00

	ATP-bound mHsp60 double-ring (EMDB 54901) (PDB 9SHJ)	ADP-bound mHsp60 single-ring (EMDB 54902) (PDB 9SHK)	ATP-bound mHsp60 single-ring (EMDB 54903) (PDB 9SHL)
**Data collection and processing**			
Magnification	105000	105000	105000
Voltage (kV)	300	300	300
Electron exposure (e–/Å^2)	50	50	50
Defocus range (μm)	0.8-1.6	0.8-1.6	0.8-1.6
Pixel size (Å)	0.8238	0.8238	0.8238
Symmetry imposed	D7	C7	C7
Initial particle images (no.)	12338206	12338206	12338206
Final particle images (no.)	143570	288807	340258
Map resolution (Å)	2.18	2.91	2.50
FSC threshold	0.143	0.143	0.143
Map resolution range (Å)	1.978 - 34.455	1.768 - 9.351	2.147 - 24.030
**Refinement**			
Initial model used (PDB code)	9ES2	9ES1	9ES2
Model resolution (Å)	2.3	3.3	2.8
FSC threshold	0.5	0.5	0.5
Map sharpening *B* factor (Å^2)	-58.0	-106.0	-97.6
Model composition			
Non-hydrogen atoms	56446	27629	28015
Protein residues	7378	3682	3689
Ligands	42	14	21
B factors (Å^2)			
Protein	101.83	9.31	72.59
Ligand	23.53	2.57	11.03
R.m.s. deviations			
Bond lengths (Å)	0.003	0.004	0.004
Bond angles (°)	0.597	0.668	0.659
Validation			
MolProbity score	1.25	1.64	1.38
Clashscore	4.76	10.15	5.69
Poor rotamers (%)	0.00	0.00	0.00
Ramachandran plot			
Favored (%)	98.19	97.44	97.66
Allowed (%)	1.81	2.56	2.34
Disallowed (%)	0.00	0.00	0.00

Cryo-EM data collection, refinement and validation statistics

Table S3.

mHsp60 single-ring subtomogram map	RMSD (all pairs; Å)		
	ATP-bound (this study)	ADP-bound (this study)	Apo (9ES3)
Global	9.9	3.4	6.0
No SP density	9.9	3.5	5.8
SP-bound	9.3	3.4	6.3

RMSD comparison between mHsp60 single-ring atomic models before and after MDFF into *in situ* mHsp60 single-ring subtomogram maps. RMSD calculations were performed using MAXIMOBY/MOBY, considering all common Cα atoms between the models for alignment and RMSD calculation.
